# Genome-wide analysis of parent-of-origin interaction effects with environmental exposure (PoOxE): An application to European and Asian cleft palate trios

**DOI:** 10.1371/journal.pone.0184358

**Published:** 2017-09-12

**Authors:** Øystein A. Haaland, Astanand Jugessur, Miriam Gjerdevik, Julia Romanowska, Min Shi, Terri H. Beaty, Mary L. Marazita, Jeffrey C. Murray, Allen J. Wilcox, Rolv T. Lie, Håkon K. Gjessing

**Affiliations:** 1 Department of Global Public Health and Primary Care, University of Bergen, Bergen, Norway; 2 Department of Genetics and Bioinformatics, Norwegian Institute of Public Health (NIPH), Oslo, Norway; 3 Centre for Fertility and Health (CeFH), Norwegian Institute of Public Health, Oslo, Norway; 4 Biostatistics and Computational Biology Branch, National Institute of Environmental Health Sciences (NIH/NIEHS), Durham, North Carolina, United States of America; 5 Department of Epidemiology, School of Public Health, Johns Hopkins University, Baltimore, Maryland, United States of America; 6 Center for Craniofacial and Dental Genetics, Department of Oral Biology, School of Dental Medicine, University of Pittsburgh, Pittsburgh, Pennsylvania, United States of America; 7 Department of Pediatrics, University of Iowa, Iowa City, Iowa, United States of America; 8 Epidemiology Branch, National Institute of Environmental Health Sciences (NIH/NIEHS), Durham, North Carolina, United States of America; 9 Department of Health Registries, Norwegian Institute of Public Health, Oslo, Norway; Yale School of Public Health, UNITED STATES

## Abstract

Cleft palate only is a common birth defect with high heritability. Only a small fraction of this heritability is explained by the genetic variants identified so far, underscoring the need to investigate other disease mechanisms, such as gene-environment (GxE) interactions and parent-of-origin (PoO) effects. Furthermore, PoO effects may vary across exposure levels (PoOxE effects). Such variation is the focus of this study. We upgraded the R-package Haplin to enable direct tests of PoOxE effects at the genome-wide level. From a previous GWAS, we had genotypes for 550 case-parent trios, of mainly European and Asian ancestry, and data on three maternal exposures (smoking, alcohol, and vitamins). Data were analyzed for Europeans and Asians separately, and also for all ethnicities combined. To account for multiple testing, a false discovery rate method was used, where q-values were generated from the p-values. In the Europeans-only analyses, interactions with maternal smoking yielded the lowest q-values. Two SNPs in the ‘Interactor of little elongation complex ELL subunit 1’ (*ICE1*) gene had a q-value of 0.14, and five of the 20 most significant SNPs were in the ‘N-acetylated alpha-linked acidic dipeptidase-like 2’ (*NAALADL2*) gene. No evidence of PoOxE effects was found in the other analyses. The connections to *ICE1* and *NAALADL2* are novel and warrant further investigation. More generally, the new methodology presented here is easily applicable to other traits and exposures in which a family-based study design has been implemented.

## Introduction

With a prevalence of 0.5 per 1000 live births, cleft palate only (CPO) is a common birth defect in humans [1, 2]. It is broadly categorized according to whether it occurs as an isolated defect or together with additional congenital anomalies. In this paper, we focus on isolated CPO.

The particularly high heritability and recurrence risk of orofacial clefts [3–8] have spurred long-standing efforts to identify genetic variants controlling risk to these common birth defects. However, as with most other complex traits, the genetic variants identified thus far explain only a small fraction of the total heritability and familial recurrence, underscoring the need to examine etiologic mechanisms beyond simple child effects alone. One alternative is to investigate the effect of a risk-allele or haplotype based on whether it is inherited from the mother or the father (i.e., parent-of-origin (PoO) effects). A difference in effect by parent of origin could occur, for example, with genes that are subject to genomic imprinting [9], which occurs when the allele from one parent is silenced but the allele from the other parent is expressed. This possibility is especially relevant for perinatal disorders because the mother defines the prenatal environment of the fetus.

Another popular approach is to explore the role of environmental factors, either independently or in combination with specific genetic variants (GxE effects). Although animal models have long demonstrated that environmental factors are important in clefting (reviewed in [10, 11]), the evidence from human studies is less conclusive. Among a wide array of environmental factors, maternal periconceptional smoking has been consistently associated with increased risk of clefting [12–14]. Since most environmental factors are modifiable, identifying GxE effects may help to target genetically susceptible subgroups of the population. A third, yet unexplored approach is to study PoO effects in interaction with environmental exposures (PoOxE); i.e., whether PoO effects vary according to the exposure status of the fetus. With the notable exception of Wang et al. (2011) [15], who assessed differential imprinting across environmental exposures in childhood asthma, the literature on PoOxE effect estimation is sparse. To address this gap, we have developed a comprehensive and user-friendly methodology that is not restricted by assumptions pertaining to imprinting. The theoretical foundation for these new methods has been presented by Skare et al. (2012) [14] and Gjerdevik et al. (2017) [16], and the methods themselves are available in the R-package Haplin [17]. The mathematics behind the PoOxE analyses is outlined in Materials and methods.

This study is based on the case-parent trio study design, which is applicable to a wide range of etiologic scenarios pertinent to perinatal disorders [18]. We had GWAS data as well as information on periconceptional exposures from the mother (cigarette smoking, alcohol intake, vitamin use) and ethnicity (European, Asian, other) for the largest collection of CPO trios to date [19]. Our aim is to identify PoOxE effects in this data set.

## Results

**Fig 1:**
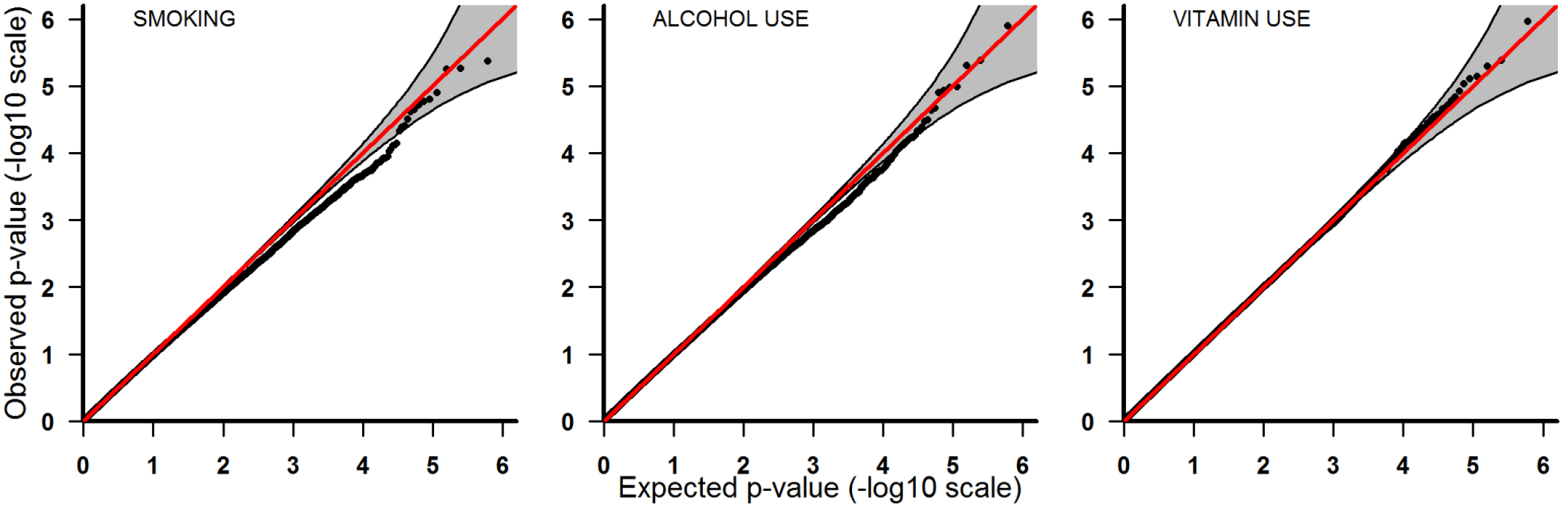
Pooled analyses of all ethnicities combined. From left to right: smoking, alcohol intake, and vitamin use.

**Fig 2:**
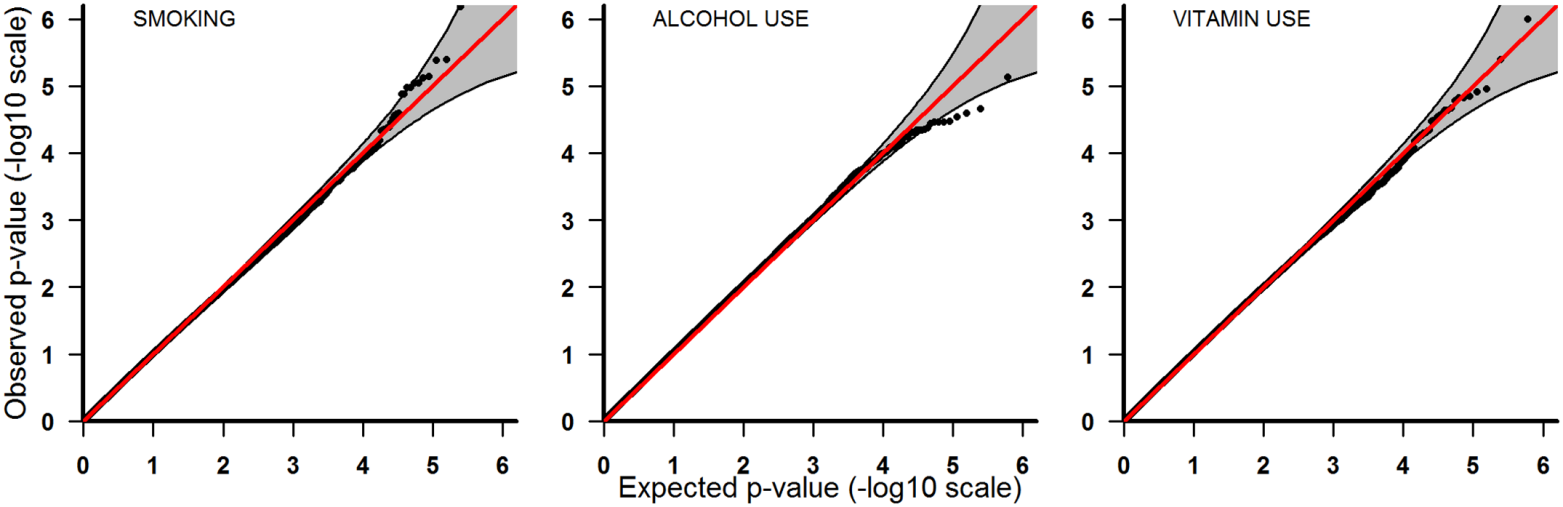
Analyses of the European sample. From left to right: smoking, alcohol intake, and vitamin use.

**Fig 3:**
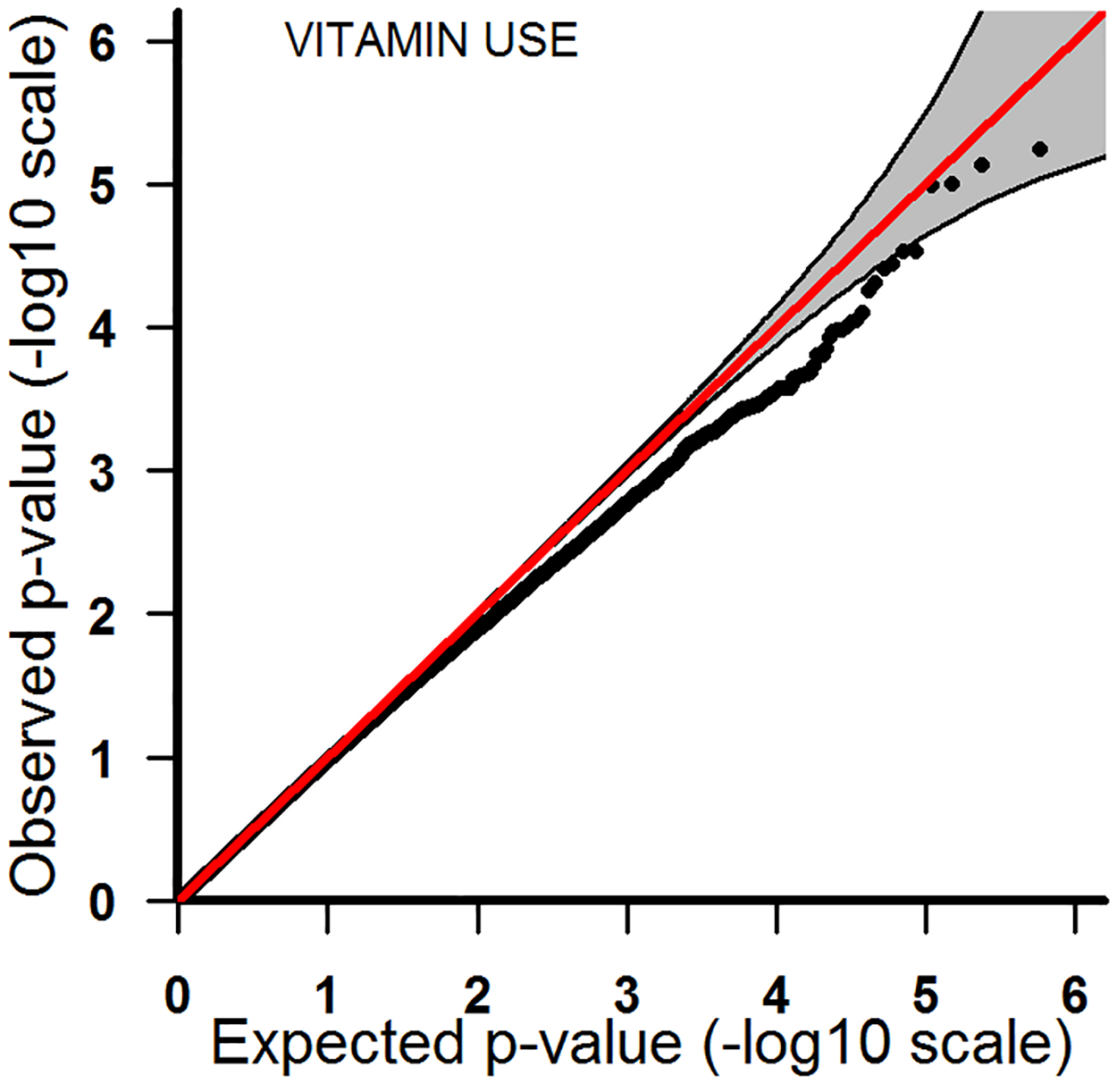
Analyses of vitamin use in the Asian sample.

We conducted three sets of analyses: pooled analyses including all participants; analyses restricted to Europeans only; and analyses restricted to Asians only. The remaining ethnic groups in our data set were too small to justify separate analyses (Table 1). Given the phenotypic consistency in clefting across ethnicities, it is reasonable to assume that a proportion of the causal variants for clefting is shared across all ethnicities. Accordingly, we present the results of the pooled analyses first, followed by the Europeans-only and Asians-only analyses. The combination of three environmental exposures and the above subgroup analyses yielded a large amount of results. For simplicity, we chose to focus on the top 20 SNPs (sorted by observed p-value) from each analysis. Details about these SNPs, including relative risk ratios (RRRs), are provided in Table 2 and Fig 1, Table 3 and Fig 2, and Table 4 and Fig 3. The corresponding Manhattan plots are provided as supplementary online material (S1 to S3 Figs). Table 5 contains the full names of all the genes mentioned in Tables 2 to 4.

**Table 1:** Population distribution according to ethnicity and trio completeness.

Ethnicity	Complete trios	Incomplete trios	Total
All	466	84	550
European	215	54	269
Asian	231	22	253
Other*	20	8	28

Columns show the number of families.

*Separate analyses were not conducted for this group.

**Table 2:** Top 20 SNPs sorted according to p-value in the pooled analysis.

	SNP ^a^	P-value	Q-value	RRR (95% CI)	Gene symbol ^b^	Shared ^c^
**SMOKING**	rs1116099	4.3e-06	0.8	7.3 (3.1–17)	NC	
	rs2964137	5.5e-06	0.8	6.9 (3.0–16)	*~ICE1*	Europe
	rs2964447	5.6e-06	0.8	6.8 (3.0–16)	*~ICE1*	Europe
	rs1348692	1.26e-05	1	6.3 (2.8–15)	NC	
	rs17401797	1.6e-05	1	0.17 (0.08–0.38)	*~ICA1/ GLCCI1*	Europe
	rs247820	1.72e-05	1	0.15 (0.06–0.36)	*ATP2C2*	
	rs6764422	1.94e-05	1	10.5 (3.6–31)	*NAALADL2*	Europe
	rs4243412	2.26e-05	1	10.2 (3.5–30)	*NAALADL2*	Europe
	rs4695808	2.45e-05	1	0.18 (0.08–0.40)	NC	
	rs10936861	3.12e-05	1	7.3 (2.9–19)	*NAALADL2*	Europe
	rs4884814	3.91e-05	1	5.4 (2.4–12)	NC	
	rs11706760	4.17e-05	1	9.5 (3.2–28)	*NAALADL2*	Europe
	rs4868953	4.68e-05	1	5.4 (2.4–12)	NC	
	rs10228906	7.24e-05	1	0.18 (0.08–0.42)	*STEAP1B*	
	rs1889706	7.59e-05	1	0.16 (0.06–0.39)	*NRG3*	
	rs2861624	7.77e-05	1	5.1 (2.3–12)	NC	
	rs6781659	8.85e-05	1	6.5 (2.5–17)	*NAALADL2*	Europe
	rs1035631	9.63e-05	1	0.20 (0.09–0.45)	*~WIF1*	
	rs13078307	0.0001146	1	0.10 (0.03–0.32)	*CNTN4*	
	rs7997369	0.0001181	1	4.9 (2.2–11)	NC	
**ALCOHOL**	rs6092934	1.3e-06	0.5	6.7 (3.1–15)	NC	Europe
	rs2587888	4.1e-06	0.7	0.19 (0.10–0.39)	*GNAO1*	
	rs12901536	5e-06	0.7	5.3 (2.6–11)	NC	
	rs16991645	1.02e-05	0.8	0.18 (0.08–0.38)	*PSMF1*	
	rs1884511	1.07e-05	0.8	0.18 (0.08–0.38)	NC	
	rs1396176	1.16e-05	0.8	0.18 (0.08–0.38)	NC	
	rs11595656	1.26e-05	0.8	0.11 (0.04–0.30)	*GRID1*	Europe
	rs12613026	2.13e-05	1	4.2 (2.2–8.1)	*HAAO*	
	rs10200371	2.32e-05	1	0.21 (0.11–0.44)	NC	
	rs12417042	3.22e-05	1	0.19 (0.09–0.42)	*GALNT18*	
	rs2560294	3.41e-05	1	0.21 (0.10–0.44)	NC	
	rs7992498	4.15e-05	1	0.23 (0.12–0.47)	NC	
	rs1451991	4.68e-05	1	0.17 (0.07–0.40)	NC	
	rs13418113	4.73e-05	1	0.19 (0.09–0.42)	NC	
	rs4794556	5.47e-05	1	0.13 (0.05–0.35)	NC	
	rs4910320	5.87e-05	1	0.20 (0.09–0.43)	*GALNT18*	
	rs4905741	5.9e-05	1	0.25 (0.12–0.49)	NC	
	rs10464419	6.26e-05	1	0.22 (0.11–0.46)	*DPP6*	
	rs9862003	6.5e-05	1	6.1 (2.5–15)	*FHIT*	
	rs4756930	6.84e-05	1	4.3 (2.1–8.7)	*SAAL1*	
**VITAMIN**	rs2830634	1.1e-06	0.5	4.8 (2.6–9.0)	NC	
	rs10087070	4.2e-06	0.7	0.15 (0.07–0.34)	NC	
	rs10087643	5e-06	0.7	0.16 (0.07–0.35)	NC	
	rs7245039	7.3e-06	0.7	0.26 (0.14–0.46)	NC	Asia
	rs11659340	7.8e-06	0.7	0.25 (0.13–0.46)	NC	
	rs2908907	9.3e-06	0.7	0.26 (0.14–0.47)	NC	Asia
	rs13099091	1.19e-05	0.7	19 (5.2–72)	NC	
	rs9874470	1.48e-05	0.7	0.25 (0.14–0.47)	*LSAMP*	
	rs11787235	1.69e-05	0.7	0.17 (0.08–0.39)	NC	
	rs1918367	1.94e-05	0.7	0.27 (0.15–0.49)	NC	Europe
	rs163474	2.14e-05	0.7	0.26 (0.14–0.48)	*ZNF659*	
	rs6024956	2.2e-05	0.7	0.27 (0.15–0.50)	NC	
	rs8087079	2.63e-05	0.7	0.27 (0.15–0.50)	NC	Asia
	rs34646750	2.72e-05	0.7	0.28 (0.15–0.51)	NC	
	rs4831129	2.93e-05	0.7	0.26 (0.14–0.49)	*LSAMP*	
	rs9947198	2.94e-05	0.7	3.7 (2.0–6.9)	NC	
	rs1026791	3.1e-05	0.7	3.5 (2.0–6.4)	*~IL22*	
	rs10960072	3.44e-05	0.7	0.25 (0.13–0.48)	NC	
	rs3214002	3.6e-05	0.7	3.8 (2.0–7.0)	NC	
	rs6593445	3.9e-05	0.7	0.23 (0.12–0.46)	NC	

^a^ SNP location according to the 1000 Genomes browser (Phase 3; https://www.ncbi.nlm.nih.gov/variation/tools/1000genomes)

^b^ NC: Not close to any known gene (at least within a 30 kb-distance). Pseudogenes and non-coding RNA (ncRNA) are excluded. ~: located within 30 kb of a gene

^c^ Shared: Also among the top 20 SNPs in either the Asians-only or the Europeans-only analyses.

**Table 3:** Top 20 SNPs sorted according to p-value in the Europeans-only analysis.

	SNP^a^	P-value	Q-value	RRR (95% CI)	Gene symbol ^b^	Shared ^c^
**SMOKING**	rs2964447	6e-07	0.14	0.09 (0.04–0.23)	*~ICE1*	Pooled
	rs2964137	7e-07	0.14	0.09 (0.04–0.23)	*~ICE1*	Pooled
	rs4243412	4.1e-06	0.4	17 (5.0–56)	*NAALADL2*	Pooled
	rs6764422	4.1e-06	0.4	17 (5.0–56)	*NAALADL2*	Pooled
	rs6771026	7.2e-06	0.4	11 (3.8–31)	NC	
	rs10936861	7.6e-06	0.4	12 (4.0–36)	*NAALADL2*	Pooled
	rs12678499	9e-06	0.4	0.08 (0.03–0.24)	*OXR1*	
	rs12548886	9.1e-06	0.4	0.08 (0.03–0.24)	*OXR1*	
	rs9661728	1.07e-05	0.4	8.1 (3.2–20)	NC	
	rs11706760	1.07e-05	0.4	15 (4.5–49)	*NAALADL2*	Pooled
	rs17401797	1.32e-05	0.5	0.13 (0.05–0.32)	*~ICA1/GLCCI1*	Pooled
	rs7545940	1.34e-05	0.5	0.11 (0.04–0.30)	*MORN1*	
	rs6454237	2.57e-05	0.8	0.11 (0.04–0.30)	*FAM46A*	
	rs10777647	2.63e-05	0.8	0.15 (0.06–0.36)	NC	
	rs9344208	2.75e-05	0.8	0.11 (0.04–0.30)	NC	
	rs12620896	3.05e-05	0.8	0.09 (0.03–0.27)	NC	
	rs17367409	3.59e-05	0.9	7.7 (2.9–20)	*ZHX2*	
	rs6781659	4.15e-05	0.9	9.9 (3.3–29)	*NAALADL2*	Pooled
	rs9449357	4.29e-05	0.9	0.12 (0.04–0.33)	NC	
	rs9344210	4.44e-05	0.9	0.12 (0.04–0.33)	NC	
**ALCOHOL**	rs6092934	7.5e-06	0.8	8.0 (3.2–20)	NC	Pooled
	rs738261	2.2e-05	0.8	5.6 (2.5–12)	*BPIFC*	
	rs10464419	2.56e-05	0.8	0.15 (0.06–0.36)	*DPP6*	Pooled
	rs1563231	2.9e-05	0.8	0.09 (0.03–0.28)	NC	
	rs13016127	3.37e-05	0.8	0.18 (0.08–0.41)	NC	
	rs760150	3.5e-05	0.8	10 (3.4–31)	*PCP4*	
	rs2271986	3.51e-05	0.8	24 (5.2–105)	*NOS1*	
	rs9658570	3.51e-05	0.8	23 (5.2–105)	*NOS1*	
	rs11595656	3.68e-05	0.8	0.10 (0.03–0.29)	*GRID1*	Pooled
	rs329138	4.3e-05	0.8	0.18 (0.08–0.41)	*~CLDN18/DZIP1L*	
	rs10498066	4.52e-05	0.8	6.7 (2.7–17)	NC	
	rs6469548	4.53e-05	0.8	0.09 (0.03–0.29)	NC	
	rs514898	4.62e-05	0.8	6.7 (2.7–18)	NC	
	rs10035580	4.63e-05	0.8	6.4 (2.6–16)	*FAM134B*	
	rs7605568	4.77e-05	0.8	6.7 (2.7–17)	NC	
	rs11076452	4.88e-05	0.8	0.18 (0.08–0.41)	NC	
	rs6848313	4.97e-05	0.8	0.18 (0.08–0.42)	*PPARGC1A*	
	rs7141416	5.1e-05	0.8	12 (3.5–38)	NC	
	rs4938094	5.71e-05	0.8	0.16 (0.06–0.39)	NC	
	rs17102505	5.77e-05	0.8	0.06 (0.02–0.24)	NC	
**VITAMIN**	rs1400316	1e-06	0.4	10 (4.0–26)	*DLG2*	
	rs881029	4.1e-06	0.8	12 (4.2–36)	*GPC1*	
	rs10933973	1.1e-05	0.8	7.5 (3.1–19)	*GUCA1C*	
	rs1290620	1.25e-05	0.8	0.14 (0.06–0.34)	*CYP4F3*	
	rs759998	1.45e-05	0.8	0.14 (0.06–0.34)	*CYP4F3*	
	rs2144410	1.52e-05	0.8	0.12 (0.04–0.31)	*TBC1D22A*	
	rs2275256	1.52e-05	0.8	9.7 (3.5–27)	*BNC2*	
	rs1918367	1.67e-05	0.8	0.13 (0.05–0.33)	NC	Pooled
	rs7033512	2.17e-05	0.8	9.1 (3.3–25)	*BNC2*	
	rs10898166	2.34e-05	0.8	0.12 (0.04–0.32)	*DLG2*	
	rs11233774	2.34e-05	0.8	0.12 (0.04–0.32)	*DLG2*	
	rs17051378	2.62e-05	0.8	10 (3.4–30)	*ANXA5*	
	rs10932619	2.75e-05	0.8	6.6 (2.7–16)	NC	
	rs722097	2.94e-05	0.8	6.8 (2.8–17)	*NINJ2*	
	rs921171	3.3e-05	0.8	6.7 (2.7–17)	*GUCA1C*	
	rs751873	3.3e-05	0.8	7.6 (2.9–20)	*SYNJ2*	
	rs739012	3.4e-05	0.8	0.13 (0.05–0.34)	*TBC1D22A*	
	rs2683045	4.54e-05	0.96	0.15 (0.06–0.38)	*CYP4F3*	
	rs4799646	4.67e-05	0.96	11 (3.4–34)	NC	
	rs1838454	4.93e-05	0.96	7.7 (2.9–20)	NC	

^a^ SNP location according to the 1000 Genomes browser (Phase 3; https://www.ncbi.nlm.nih.gov/variation/tools/1000genomes)

^b^ NC: Not close to any known gene (at least within a 30 kb-distance). Pseudogenes and non-coding RNA (ncRNA) are excluded. ~: located within 30 kb of a gene

^c^ Shared: Also among the top 20 SNPs in either the Asians-only or the pooled analyses.

**Table 4:** Top 20 SNPs sorted according to p-value in the Asians-only analysis.

SNP ^a^	P	Q	RRR (95% CI)	Gene symbol ^b^	Shared ^c^
rs12519078	5.8e-06	1	12 (4.0–33)	NC	
rs1345405	7.5e-06	1	12 (4.1–36)	NC	
rs11859629	1.02e-05	1	11 (3.9–34)	NC	
rs2052509	1.03e-05	1	0.06 (0.02–0.21)	*TENM2*	
rs7499215	2.99e-05	1	10 (3.5–31)	NC	
rs2908915	3.01e-05	1	0.11 (0.04–0.32)	NC	
rs2908907	3.68e-05	1	0.11 (0.04–0.32)	NC	Pooled
rs2964356	3.97e-05	1	0.11 (0.04–0.32)	NC	
rs1996644	4.99e-05	1	0.10 (0.03–0.30)	NC	
rs8087079	5.66e-05	1	0.10 (0.03–0.31)	NC	Pooled
rs6683070	7.91e-05	1	0.11 (0.04–0.33)	NC	
rs7974646	9.1e-05	1	0.11 (0.03–0.33)	NC	
rs13119549	9.29e-05	1	7.9 (2.8–22)	*~HELT*	
rs12960489	9.89e-05	1	0.11 (0.04–0.33)	NC	
rs2303447	0.0001042	1	0.12 (0.04–0.35)	*TPD52*	
rs2098898	0.0001042	1	0.12 (0.04–0.35)	*TPD52*	
rs1337161	0.0001073	1	0.12 (0.04–0.35)	NC	
rs1252951	0.00012	1	0.13 (0.04–0.36)	*~MIS18BP1*	
rs2289487	0.0001416	1	26 (4.8–141)	*PLIN1*	
rs7245039	0.0001565	1	0.12 (0.04–0.36)	NC	Pooled

^a^ SNP location according to the 1000 Genomes browser (Phase 3; https://www.ncbi.nlm.nih.gov/variation/tools/1000genomes)

^b^ NC: Not close to any known gene (at least within a 30 kb-distance). Pseudogenes and non-coding RNA (ncRNA) are excluded. ~: located within 30 kb of a gene

^c^ Shared: Also among the top 20 SNPs in either the pooled or Europeans-only analyses.

**Table 5:** Full gene names*.

Smoking	Alcohol use	Vitamin use
*ATP2C2*: ATPase secretory pathway Ca2+ transporting 2	*BPIFC*: BPI fold containing family C	*ANXA5*: Annexin A5
*CNTN4*: Contactin 4	*CLDN18*: Claudin 18	*BNC2*: Basonuclin 2
*FAM46A*: Family with sequence similarity 46 member A	*DPP6*: Dipeptidyl peptidase like 6	*CYP4F3*: Cytochrome P450 family 4 subfamily F member 3
*GLCCI1*: Glucocorticoid induced 1	*DZIP1L*: DA2 introducing protein	*DLG2*: Discs large MAGUK scaffold protein 2
*ICA1*: Islet cell autoantigen 1	*FAM134B*: Family with sequence similarity 134 member B	*GPC1*: Glypican 1
ICE1: Interactor of little elongation complex ELL subunit 1	*FHIT*: Fragile histidine triad	*GUCA1C*: Guanylate cyclase activator 1C
*MORN1*: MORN repeat containing 1	*GALNT18*: Polypeptide N-acetylgalactosaminyltransferase 18	*HELT*: Helt bHLH transcription factor
*NAALADL2*: N-acetylated alpha-linked acidic dipeptidase like 2	*GNAO1*: G protein subunit alpha o1	*IL22*: interleukin 22
*NRG3*: Neuregulin 3	*GRID1*: Glutamate ionotropic receptor delta type subunit 1	*LSAMP*: Limbic system associated membrane protein
*OXR1*: Oxidation resistance 1	*HAAO*: 3-hydroxyanthranilate 3,4-dioxygenase	*MIS18BP1*: MIS18 binding protein 1
*STEAP1B*: STEAP family member 1B	*NOS1*: Nitric oxide synthase 1	*NINJ2*: Ninjurin 2
*WIF1*: WNT inhibitory factor 1	*PCP4*: Purkinje cell protein 4	*PLIN1*: Perilipin 1
*ZHX2*: Zinc fingers and homeoboxes 2	*PSMF1*: Proteasome inhibitor subunit 1	*SYNJ2*: Synaptojanin 2
	*PPARGC1A*: PPARG coactivator 1 alpha	*TBC1D22A*: TBC1 domain family member 22A
	*SAAL1*: Serum amyloid A like 1	*TENM2*: Teneurin transmembrane protein 2
		*TPD52*: Tumor protein D52
		*ZNF659*: Zinc finger protein 659

*The full gene names were retrieved from the NCBI Entrez Gene Database (https://www.ncbi.nlm.nih.gov/gene).

Gene IDs used in Tables 2 to 4

To adjust for multiple testing, we used a false discovery rate method where q-values are calculated from observed p-values [20]. We used a q-value of 0.1 to assess statistical significance, which means that at least 90% of the significant SNPs are expected to be true positives. Across all analyses, several SNPs had q-values ranging from 0.1 to 0.5 (Tables 2 to 4). This corresponds to a false discovery rate between 10% and 50%, implying that many of these SNPs are potentially associated with PoOxE effects. Fig 1 shows QQ-plots for the pooled analyses, comprising all ethnicities. All of the most significant SNPs are within the 95% confidence band at the upper right corner of the distribution. The lowest q-values were 0.8 for rs1116099 for maternal smoking, 0.5 for rs6092934 for maternal alcohol intake, and 0.5 for rs2830634 for maternal vitamin use (Table 2).

QQ-plots for the Europeans-only analyses are shown in Fig 2. The plot for smoking is particularly notable because all the top 12 SNPs had lower p-values than expected, even though most of them were located within the 95% confidence band. Specific p-values and q-values for these SNPs are provided in Table 3. All of these q-values were below 0.5 for the top 12 SNPs, but markedly higher for the remaining SNPs. Among these 12 SNPs, both rs2964447 and rs2964137 had a q-value of 0.14 (RRR = 0.09, 95% CI: 0.04–0.23). For alcohol intake and vitamin use, the top SNPs were rs6092934 (q = 0.8, RRR = 8.0, 95% CI: 3.2–19.8) and rs1400316 (q = 0.4, RRR = 10.1, 95% CI: 4.0–25.6), respectively.

The Asians-only analyses were uninformative due to the low number of trios in which the mother had smoked or consumed alcohol (Table 6). Consequently, tests for interaction had less power than the other analyses. For vitamin use, the QQ-plot did not deviate appreciably from the expected pattern (Fig 3). Table 4 shows the p-values and q-values for the top 20 SNPs. All the SNPs in the Asians-only analyses had q-values equal to one.

**Table 6:** Characteristics of maternal exposures according to ethnicity.

Ethnic group	Maternal exposure	No	Yes	Missing
All* (n = 550)	Smoking	463	86	1
	Vitamin	265	224	61
	Alcohol	423	122	5
European (n = 269)	Smoking	195	74	0
	Vitamin	88	155	26
	Alcohol	160	108	1
Asian (n = 253)	Smoking	245	8	0
	Vitamin	170	51	32
	Alcohol	241	9	3

*Includes ethnicities that are not European or Asian

**Fig 4:**
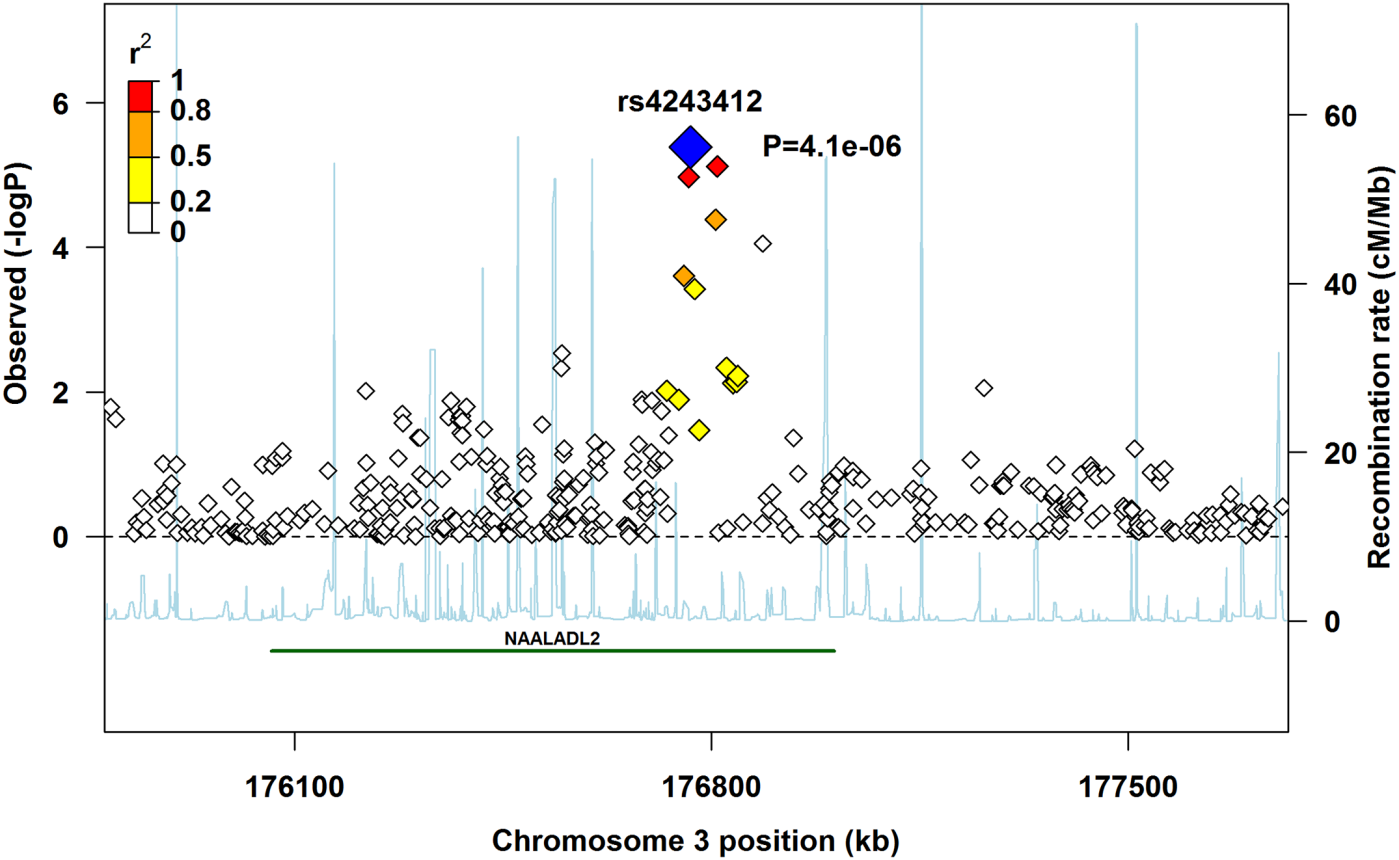
Regional association plot for rs4243412 in *NAALADL2*. The lead SNP is shown in blue, with its associated p-value.

**Fig 5:**
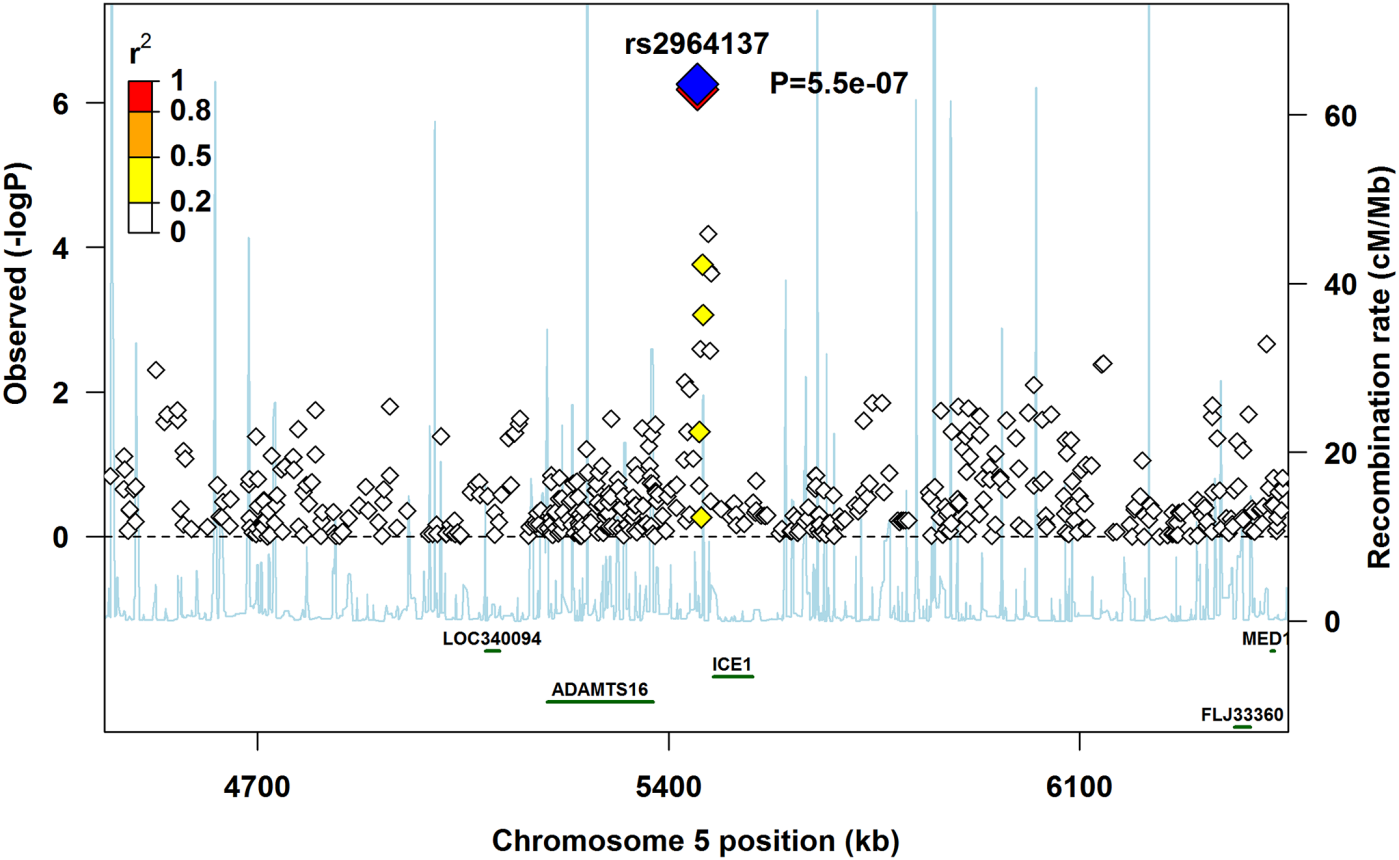
Regional association plot for rs2964137 near *ICE1*. The lead SNP is shown in blue, with its associated p-value.

Several of the top 20 SNPs were the same across the three main analyses (pooled, Europeans-only, and Asians-only). The pooled and Europeans-only analyses had eight of the top SNPs in common for PoOxSmoke, three for PoOxAlcohol, and one for PoOxVitamin (Table 2). Similarly, the pooled and Asians-only analyses had three of the top SNPs in common for PoOxVitamin (Table 2). As several of the top 20 SNPs were located in the gene for ‘N-acetylated alpha-linked acidic dipeptidase-like 2’ (*NAALADL2*), we generated a regional association plot for rs4243412, which was the SNP in *NAALADL2* with the lowest p-value in the Europeans-only analysis (Fig 4). We created a similar plot for rs2964137, which was the SNP with the lowest p-value in the pooled analysis (Fig 5). This SNP is located near the ‘Interactor of little elongation complex ELL subunit 1’ (*ICE1*) gene, and was also found among the top 20 SNPs in the Europeans-only analysis (Table 2).

Because PoO effects and maternal effects may be mutually confounded [21], we performed sensitivity analyses on the above-mentioned top 20 SNPs, and adjusted for potential maternal effects in each stratum of exposure. In these analyses, the RRRs were similar to those in Tables 2 to 4, and the Bonferroni corrected p-values for the interaction between maternal and environmental effects were all equal to 1.

## Discussion

Our study used data from the largest collection of CPO trios to date [19] to investigate the hitherto untested possibility of interactions between PoO effects and maternal environmental exposures that have previously shown associations with clefts. We introduce new methodology that not only tests for PoOxE effects but also quantifies them as ratios of relative risks. All analyses were implemented in the R-package Haplin, which accommodates a wide range of etiologic scenarios suitable for family-based study designs. An example code for PoOxE analysis is provided in S1 Appendix.

### Pooled analyses

For PoOxSmoke, all p-values were higher in the pooled analyses than in the Europeans-only analyses, suggesting a dilution of effects after pooling data. This reduction of the effect estimate in the pooled analyses may reflect heterogeneity of effect among the subgroups. The opposite was true for PoOxAlcohol, which may indicate a more consistent effect of alcohol across ethnicities. Regarding maternal smoking, multiple SNPs in *NAALADL2* indicated the presence of PoOxSmoke effects. No genes or SNPs stood out in the PoOxVitamin analysis.

### Europeans-only analyses

We found suggestive evidence of a PoOxSmoke effect for rs2964137 and rs2964447. Although neither SNP is located within any known gene, both lie near *ICE1* and are only 2–15 kb from three copy-number variant regions (CNVRs). As in the pooled PoOxSmoke analysis, several top SNPs are located in *NAALADL2*. Previous analyses of data from genome rearrangements, GWAS, and gene-expression have linked this gene to various disorders, including mild retardation [22] and cancer [23]. We have not been able to find a connection between clefting and ‘Glucocorticoid induced transcript 1’ (*GLCCI1*), ‘Islet cell autoantigen 1’ (*ICA1*), or ‘Zinc finger and homeobox 2’ (*ZHX2*).

Regarding PoOxAlcohol effects, ‘Nitric oxide synthase 1’ (*NOS1*) and ‘Dipeptidyl-peptidase 6’ (*DPP6*) were among the most interesting genes. *NOS1* acts as a physiological modulator of skeletal muscle function and *DPP6* is involved in embryonic craniofacial development [24, 25]. Another member of the nitric oxide synthase family, *NOS3*, is involved in the folate pathway and has previously been linked to a higher risk of isolated CL/P in a non-Hispanic white population [26]. Furthermore, analysis of biopsies of soft palate muscle tissues from children with isolated clefts showed that NOS1 immunoreactivity in the muscle fibers was strongly influenced by the cleft itself [27].

In the PoOxVitamin analysis, three SNPs were located in the ‘Discs, large homolog 2’ (*DLG2*) gene on chromosome 11q14.1. One of these SNPs in *DLG2*, rs1400316, had the lowest q-value (0.4). Little has been reported about its role in clefting. Three other genes, ‘Guanylate cyclase activator 1C’ (*GUCA1C*), ‘TBC1 domain family, member 22A’ (*TBC1D22A*) and ‘Cytochrome P450, family 4, subfamily F, member 3’ (*CYP4F3*), each contain two of the top 20 SNPs from this analysis. Based on the literature, however, *GUCA1C* and *TBC1D22A* do not appear to have any connections to clefting. In contrast, *CYP4F3* belongs to the cytochrome P450 gene family, which is known to be involved in the biotransformation of endobiotics and xenobiotics [28], and may be relevant for clefting. Still, the q-values for SNPs in *CYP4F3* were 0.8 or higher.

### Asians-only analyses

Compared with European women, Asian women generally consume little alcohol and tobacco [29, 30], which would be expected to be even less among those who are pregnant or planning to be pregnant. This was also observed in our data (Table 6). Even though a lack of observations was not a problem for the PoOxVitamin analyses, all the q-values were equal to one and there were no convincing associations overall for this ethnic group. Regarding ethnic specificity and generalizability, none of the top SNPs in the Asians-only analyses were among the top SNPs in the Europeans-only analyses (Tables 3 and 4), which suggests ethnic-specific effects. Still, the lack of markers in common was somewhat unexpected, as GxE effects have previously been reported across the two ethnicities in the same sample population studied here [31]. However, that study used a different approach; the pooled sample was analyzed first and the top SNPs were verified to see whether the results were consistent across ethnicities. Additionally, the authors did not consider PoOxE.

### Methodological considerations

The case-parent trio study design coupled with a large data set provided an excellent opportunity to explore PoOxE effects. The study design protects against false positives due to population substructure, because it aims at detecting asymmetries in allele transmission from parents to the affected child (proband), as opposed to considering only differences in allele frequencies at a population level. Still, if populations of different ethnicities react differently to a given exposure, such that there is a PoOxE effect in one population but not in the other, this effect may be muted or even go undetected in the combined population. It is therefore judicious to stratify analysis by ethnicity.

PoO effects may be seen when a gene associated with a given phenotype is also subjected to genomic imprinting [32, 33]. Through DNA methylation, the expression of a particular gene can be upregulated or downregulated depending on its parental origin [9, 34]. It is thus reasonable to assume that maternal environmental exposures capable of influencing methylation patterns might also influence the phenotype differently for maternally and paternally inherited alleles. Hence, it is conceivable that looking specifically for PoOxE effects rather than standard PoO or GxE effects alone might increase the chance of finding gene effects that are indicative of, for instance, genomic imprinting.

While PoOxE searches combine PoO searches with ordinary GxE searches in a natural way, there is a price to pay in the form of added complexity. Nevertheless, the total PoOxE effect at a locus with two alleles and a dichotomous environmental exposure can be measured as a single ratio of relative risks (RRR). We have

$$\text{RRR}=\frac{{\text{RRR}}_{\text{PoO}}\left(1\right)}{{\text{RRR}}_{\text{PoO}}\left(0\right)}=\frac{{\text{RR}}_{\text{mat}}\left(1\right)/{\text{RR}}_{\text{pat}}\left(1\right)}{{\text{RR}}_{\text{mat}}\left(0\right)/{\text{RR}}_{\text{pat}}\left(0\right)},$$ (1)

where RR_mat_(*S*) and RR_pat_(*S*) are as explained in Materials and Methods, and RRR is the ratio of PoO effects in the two strata. If RRR > 1, the interpretation is that the PoO effect RR_mat_(1)/RR_pat_(1) in stratum 1 is larger than the corresponding RR_mat_(0)/RR_pat_(0) in stratum 0. Note that this may come about in different ways. For example, consider an allele that increases the risk only when inherited from exposed mothers, so that RR_mat_(1) > 1. Because the other RRs are equal to 1, RRR would be larger than 1. Similarly, if the allele is protective when inherited from unexposed mothers but has no effect in other situations, RR_mat_(0) < 1, and again RRR > 1. One might also observe more complex patterns, such as an increased risk when the allele is inherited from the mother, where this effect is larger among the exposed than the unexposed; that is,

$${\text{RR}}_{\text{mat}}\left(1\right)\;>{\text{ RR}}_{\text{mat}}\left(0\right){\text{ and RR}}_{\text{pat}}\left(1\right)\;={\text{ RR}}_{\text{pat}}\left(0\right),$$

and we would again have RRR > 1. The actual direction of the effect may depend on which allele and exposure group are chosen as reference, which is a general problem when assessing GxE in case-only designs.

**Fig 6:**
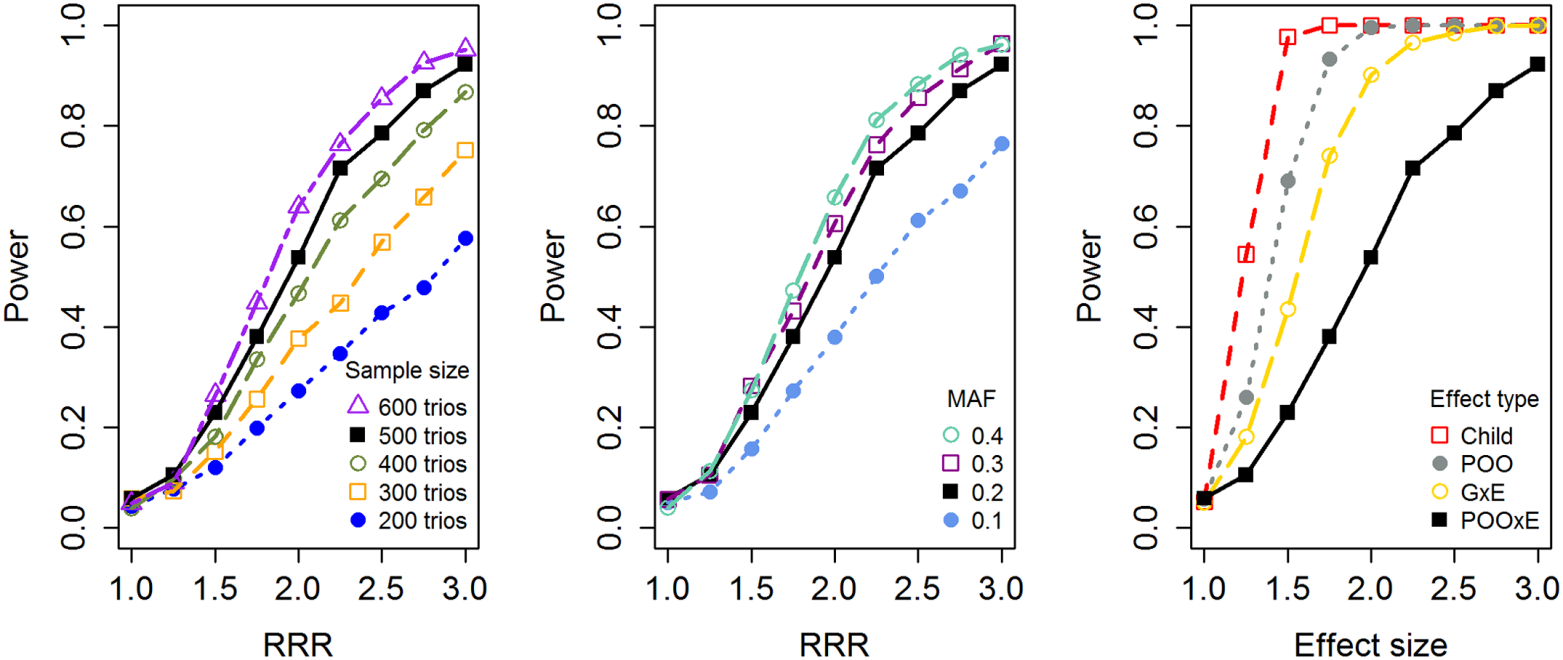
Simulation-based power curves. Left panel: Power versus relative risk ratio (RRR) for different sample sizes, minor allele frequency (MAF) = 0.2, significance level = 0.05. Middle panel: Power versus RRR for different MAFs, total sample size = 500. Right panel: Power versus RR or RRR, as applicable to each effect type, MAF = 0.2, total sample size = 500. Note that the black curve with full squares is identical in all panels (based on a total of 500 trios, MAF = 0.2, and PoOxE). In the PoOxE analysis, we have varied the RR of the maternal allele with exposure status.

While ordinary PoO analyses consider the ratio RR_mat_/RR_pat_ for both strata combined, and ordinary GxE analyses consider RR(1)/RR(0) without accounting for parental origin, the full PoOxE RRR involves comparing four quantities—the effects of maternally and paternally derived alleles computed in both strata separately. Thus, a certain loss of power would be expected relative to the standard tests for PoO and GxE effects. This is indeed what we observe in the power simulations (Fig 6, right panel). We therefore decided not to include maternal genomic effects in the full GWAS analysis, since this is likely to further reduce power to detect PoO effects [21]. Instead, we performed sensitivity analyses to remove any positive confounding from maternal effects for the 20 most promising SNPs in each set of analyses (shown in Tables 2 to 4). It is not particularly likely that any of the genes involved in the sensitivity analyses would operate through maternal effects. Complex, but less likely scenarios where maternal effects cancel out PoO effects may be missed by this approach, however.

As shown in Fig 6, PoOxE analyses will generally have lower power, given similar effect sizes, compared with PoO and GxE analyses. However, because PoOxE effects are measured as ratios of RRRs (see Eq (1)), it is hypothetically possible that PoOxE effects are larger than PoO effects or GxE effects, in particular in the presence of ‘qualitative interactions’, where effects are in opposite directions across strata. This is illustrated in S1 Appendix, and may partly explain some of the large effects in Tables 2 to 4. Under such scenarios, some of the lost power may be regained. Nevertheless, none of the q-values were lower than 0.14, which suggests that low power may have been an issue in this study. Still, several SNPs had q-values below 0.5, meaning that we expect fewer than half of them to be false positives. SNPs presented in Tables 2 to 4 should be interpreted as candidates to be further investigated in other studies. The next steps would be to replicate these candidates in other data sets/populations, followed by targeted functional analyses to help elucidate the importance of these SNPs in the interplay between environmental factors and risk of CPO.

To summarize, this study presents new methodology, implemented in the R-package Haplin, to investigate PoOxE effects in the context of family trios or duos. Our analyses pointed to several SNPs with PoOxSmoke effects in the European sample. We were unable to assess the generalizability of this finding across ethnicities, because few of the Asian mothers smoked cigarettes or consumed alcohol. We did not find any evidence for PoOxAlcohol effects in the European sample, and there were no PoOxVitamin effects in either ethnicity. Still, these analyses highlight the versatility of Haplin in studying complex disease models.

## Materials and methods

### Study participants

The majority of the participants belonged to one of two major ethnicities (European or Asian). Table 1 outlines the population distribution by ethnicity and trio completeness, and Table 6 summarizes characteristics of the maternal exposures by ethnicity.

### Quality control

Genotypes for 569 244 SNPs were available for the current analyses. The PLINK software [35] was used for quality control, with the following criteria applied for excluding SNPs: (i) >5% missing genotype for a given SNP, (ii) minor allele frequency (MAF) <5%, (iii) Hardy-Weinberg equilibrium (HWE) p-value <0.001 for parental alleles, (iv) >10% Mendelian error rate, and finally (v) linkage disequilibrium (LD) of *r*^2^ = 1 with other SNPs (to exclude SNPs with redundant information due to complete LD). Overall, genotypes for 550 families with isolated CPO were available for the current analyses. Criteria for excluding individuals were: (vi) >10% missing genotype within an individual, and (vii) >5% Mendelian errors within a family. Table 7 provides the total number of individuals after the above pruning. Because none of the families had Mendelian error rates >5%, they were all retained in the analyses. The total number of SNPs remaining after quality control is shown in Table 8, along with the different criteria used for pruning.

**Table 7:** Individuals before and after pruning.

Total individuals	1659
Genotype call rate <10%	84
Missing phenotype	217
*Remaining parents	783
*Remaining probands	575
-with two parents	475
-with one missing parent	84
-with two missing parents	16

*Remaining individuals refer to those without missing phenotype or genotype call rate <10%.

**Table 8:** SNPs before and after pruning.

Total	569244
Failed HWE-test	80566
Failed missingness test	2034
Failed SNP frequency test	57442
Mendelian errors detected	1129
*r*^2^ = 1 with flanking SNPs	6170
*Remaining SNPs	424401

*Remaining SNPs refer to those without deviations from HWE (p<0.001), more than 5% missed calls, minor allele frequencies <5%, or Mendelian errors >1%.

Genotyping rate in remaining individuals was above 0.998.

Note that a SNP may be excluded for failing more than one test criterion. Hence, the numbers do not necessarily add up.

### Statistical analysis

All analyses were conducted using the statistical software package Haplin, http://people.uib.no/gjessing/genetics/software/haplin. Haplin is particularly tailored to the analysis of offspring-parent trios and duos, but is also applicable to case-control data [17]. It is implemented as a package in the statistical programming language R [36]. We applied the function haplinSlide to analyze all SNPs sequentially. For each SNP, a log-linear maximum likelihood model is applied to the trio genotype frequencies, allowing different risk of disease (penetrance) depending on the parent of origin of the allele. The effect of each SNP was assumed to be multiplicative in allele dose, with the most common (major) allele used as reference. Missing alleles were imputed using the EM-algorithm; standard errors and p-values were corrected for this imputation [17].

The following section outlines how the PoOxE effects are computed in Haplin. First, a PoO analysis is performed for each stratum of an exposure, where *S* = 0 represents the unexposed and *S* = 1 the exposed. The PoO analysis in stratum *S* computes two relative risks

$${\text{RR}}_{\text{mat}}\left(S\right)=\frac{P\left(\text{CPO}|\text{pat}=a,\text{mat}=a_{1},S\right)}{P\left(\text{CPO}|\text{pat}=a,\text{mat}=a_{0},S\right)}$$

for a maternally inherited allele, and

$${\text{RR}}_{\text{pat}}\left(S\right)=\frac{P\left(\text{CPO}|\text{pat}=a_{1},mat=a,S\right)}{P\left(\text{CPO}|\text{pat}=a_{0},mat=a,S\right)}$$

for a paternally inherited allele, where *a*_0_ is the reference allele, *a*_1_ is the alternative allele, and “*a*” denotes any one of the two alleles. The PoO relative risk ratio (RRR_PoO_) then compares the two separate relative risks, so that

$${\text{RRR}}_{\text{PoO}}\left(S\right)=\frac{{\text{RR}}_{\text{mat}}\left(S\right)}{{\text{RR}}_{\text{pat}}\left(S\right)}.$$

RRR_PoO_ = 1 means *a*_1_ increases (or decreases) the risk by the same amount regardless of whether the allele is maternally or paternally inherited. Next, Haplin compares the RRR_PoO_ for all strata. In the case of two strata, *S* = 0 represents the unexposed and *S* = 1 the exposed, and Haplin tests whether RRR_PoO_(0) = RRR_PoO_(1). The test is performed as a Wald test by exploiting the fact that the estimated log(RRR_PoO_(0)) and log(RRR_PoO_(1)) are independent and asymptotically normally distributed, as outlined in Skare et al. (2012) [14] and Gjerdevik et al. (2017) [16].

P-values from the PoOxE analyses were displayed in a QQ-plot, with expected p-values plotted against the observed. Under the null hypothesis of no PoOxE effect, all SNPs should lie along the diagonal line representing a uniform distribution, whereas significant SNPs are expected to appear markedly above the diagonal line and outside the confidence bands.

To visualize the strength of the association signal and regional information flanking the most significant SNPs, we used a modified version of the R-script for regional plots available at http://www.broadinstitute.org/files/shared/diabetes/scandinavs/assocplot.R. The plot also displays the degree of LD between top SNPs and neighboring SNPs, recombination patterns, and positional information about genes in the region [37].

To assess the *a priori* power to detect PoOxE effects with our model, we performed power simulations based on 1000 replications and a significance level of 0.05 (Fig 6). The black line shows the power for a PoOxE analysis based on 500 case-parent trios (consistent with the sample size in this study), a MAF of 0.20, and equally-sized exposed and unexposed groups. The left panel of Fig 6 depicts different sample sizes and the middle panel depicts different MAFs. The right panel shows the power for different etiologic scenarios (child, PoO, GxE, and PoOxE). The child effect is the direct risk associated with the allele when it is carried by the child, regardless of parental origin or environmental exposures. The PoO effect is the risk associated with maternally-inherited alleles relative to paternally-inherited alleles. The GxE effect is the ratio of RRs in the two exposure groups. Finally, the PoOxE effect is the maternal to paternal risk ratio for the exposed divided by the same ratio for the unexposed.

### Ethics approvals

This specific study did not need approval from an ethics committee because ethics approvals for the consortium were obtained from the respective ethics committees at each institution contributing data to the consortium. Details have been provided in our original publication [19].

## Supporting information

S1 Fig Manhattan plots for the different exposures in the analyses of the pooled sample. SNPs with p-values below 10^−5^ are in blue.
(TIFF)

S2 Fig Manhattan plots for the different exposures in the analyses of the European sample. SNPs with p-values below 10^−5^ are in blue.
(TIFF)

S3 Fig Manhattan plots for the different exposures in the analyses of the Asian sample. (TIFF)

S1 Appendix Example code for PoOxE analysis. (DOCX)
