# Supplementary figures and images for: Genome-wide analysis of parent-of-origin interaction effects with environmental exposure (PoOxE): An application to European and Asian cleft palate trios
Source: PLoS One. 2017 Sep 12;12(9):e0184358. doi: 10.1371/journal.pone.0184358 (PMC5595425; doi:10.1371/journal.pone.0184358)

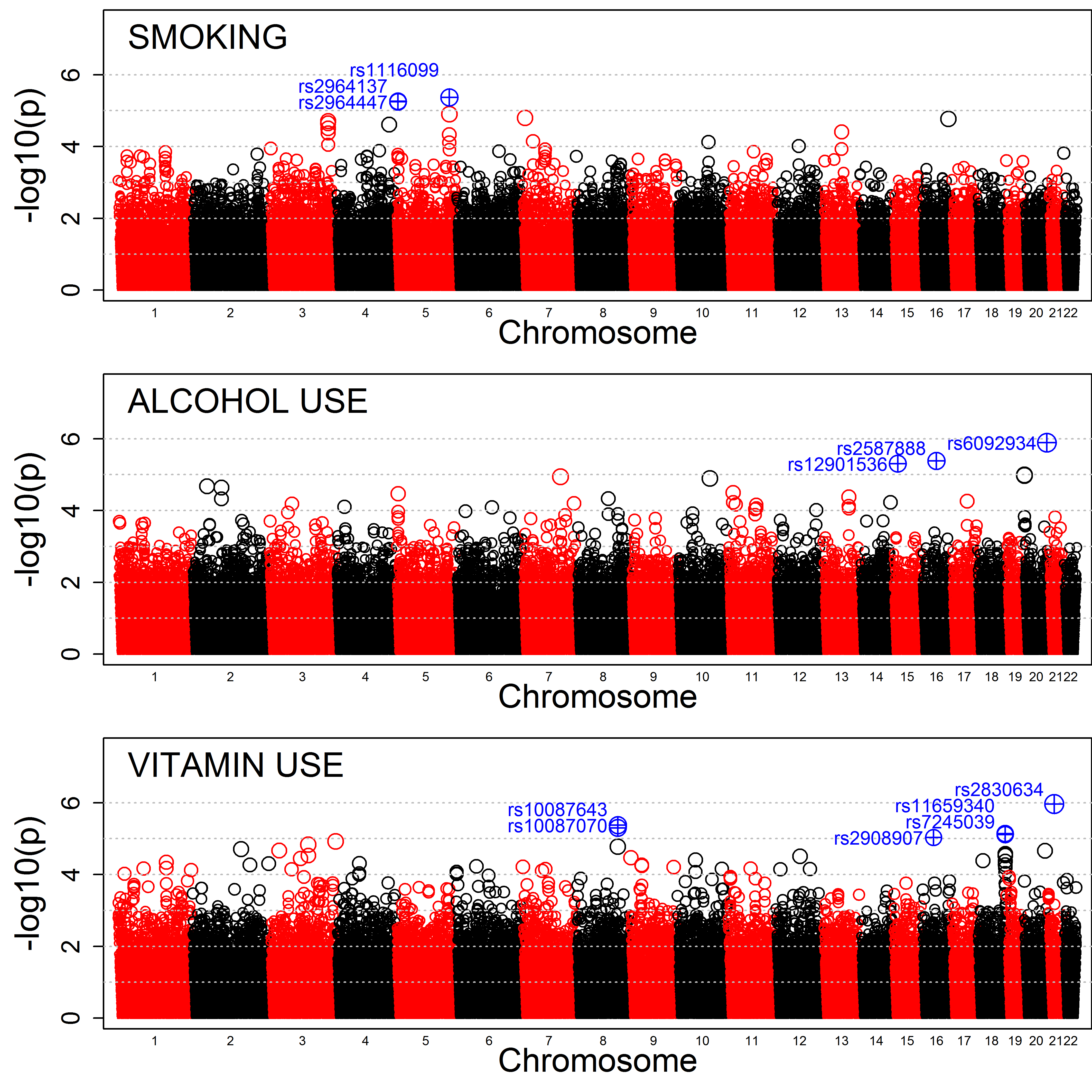

Supplement: S1 Fig — SNPs with p-values below 10−5 are in blue. (TIFF) [file pone.0184358.s001.tiff]

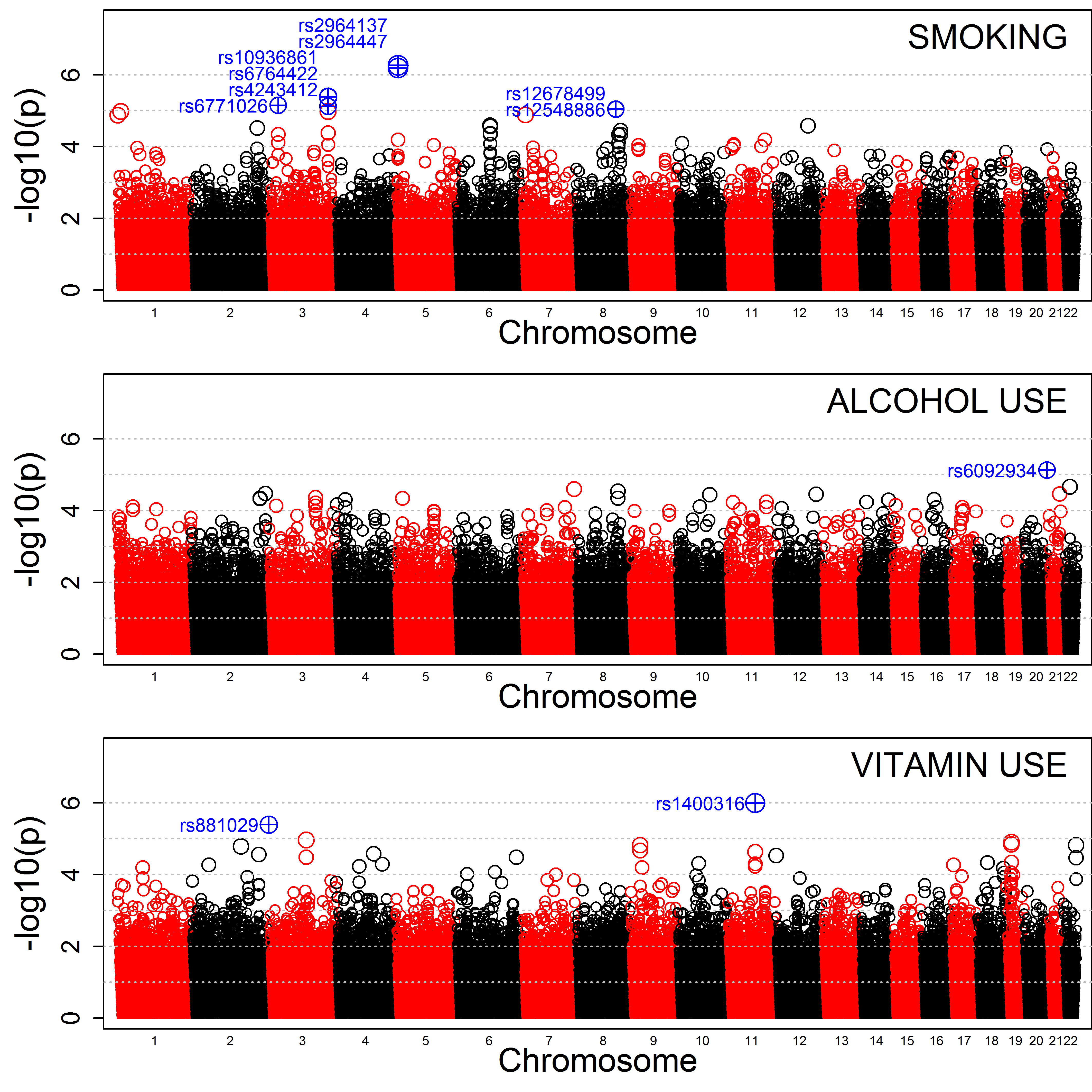

Supplement: S2 Fig — SNPs with p-values below 10−5 are in blue. (TIFF) [file pone.0184358.s002.tiff]

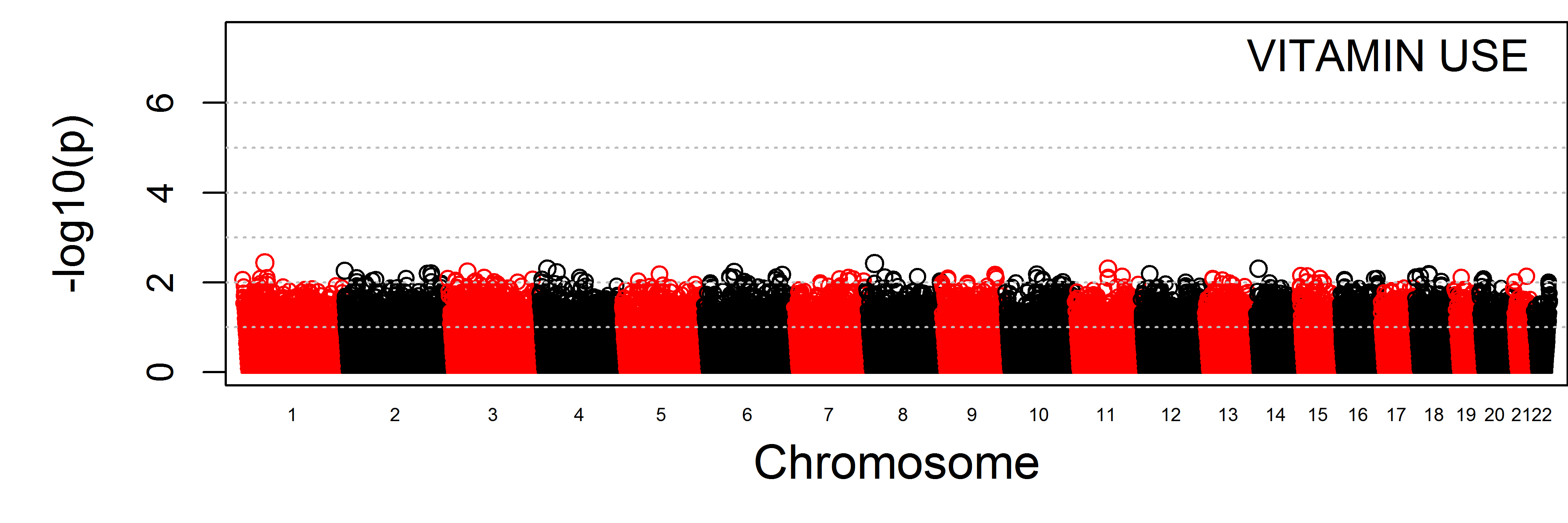

Supplement: S3 Fig — (TIFF) [file pone.0184358.s003.tiff]
