# Supplementary material for: Genome-wide analysis of parent-of-origin interaction effects with environmental exposure (PoOxE): An application to European and Asian cleft palate trios
Source: PLoS One. 2017 Sep 12;12(9):e0184358. doi: 10.1371/journal.pone.0184358 (PMC5595425; doi:10.1371/journal.pone.0184358)
Supplement: S1 Appendix — (DOCX) [file pone.0184358.s004.docx]

S1 Appendix

In order to illustrate how the new Haplin functionality works, we here use Haplin to analyze a simulated data set, which consists of one SNP genotyped in 500 case trios. The frequencies are 0.5 for both alleles, A1 and A2, and we use A2 as the reference. The RR varies across strata and parental origin as follows: A maternal A1 is detrimental in the the first stratum ($\mathrm{RR}_{\mathrm{mat}}$(1)=2) and protective in the second ($\mathrm{RR}_{\mathrm{mat}}$(2)=1/2), whereas a paternal A1 is protective in the first ($\mathrm{RR}_{\mathrm{pat}}$(1)=1/2) and detrimental in the second ($\mathrm{RR}_{\mathrm{pat}}$(2)=2). This setup ensures that the effects cancel out unless a full PoOxE analysis is run, as will be illustrated below.

Before simulating the data, we set the seed, so that all users get the exact same results if the code is run on their own computer. Then we run the code for simulating data, using the function **hapSim()**. For a thorough explanation of its arguments, please refer to the help function in R. The data set is saved to the file "sim1.dat" in the directory "sim".

library(Haplin)

set.seed(12)

hapSim(nall=2, n.strata = 2, cases=list(c(mfc=500), c(mfc=500)),
 haplo.freq = c(0.5, 0.5), RRcm=list(c(2, 1), c(1/2, 1)),
 RRcf = list(c(1/2, 1), c(2, 1)), RRstar=c(1, 1), dire="sim", n.sim=1,
 ask=F, verbose=F)

Now we use the function **haplinSlide()** to analyse the data, assuming no GxE or PoO effects.

res1 <- haplinSlide(filename = "sim/sim1.dat", markers = 1,
 winlength = 1, reference = 1, n.vars = 1,
 allele.sep = " ")

##
## Running Haplin on Window '1' (1/1)...: done
##
## --- haplinSlide has completed ---

Full results are available using res1$`1`, but we are only interested in effect estimates (RR or RRR) along with relevant p-values. They can be obtained using the following code.

signif(res1$`1`[c("RR.est.", "RR.p.value")], 3)

## RR.est. RR.p.value
## 1 1.00 NA
## 2 1.09 0.338

As expected, the estimated RR is 1.09 (p=0.338), suggesting that the SNP is not associated with the outcome. Running the analysis allowing for PoO effects (poo = T) does not change the results appreciably.

res2 <- haplinSlide(filename = "sim/sim1.dat", markers = 1,
 winlength = 1, reference = 1, n.vars = 1,
 allele.sep = " ", poo = T)

##
## Running Haplin on Window '1' (1/1)...: done
##
## --- haplinSlide has completed ---

signif(res2$`1`[c("RRcm.est.", "RRcm.p.value", "RRcf.est.", "RRcf.p.value",
 "RRcm_RRcf.est.", "RRcm_RRcf.p.value")], 3)

## RRcm.est. RRcm.p.value RRcf.est. RRcf.p.value RRcm_RRcf.est.
## 1 1.00 NA 1.00 NA 1.0
## 2 1.03 0.759 1.15 0.171 0.9
## RRcm_RRcf.p.value
## 1 NA
## 2 0.286

The maternal A1 has an estimated RR of 1.03 (p=0.759), and the paternal an estimated RR of 1.15 (p=0.171). Overall, RRR=$1.03/1.15\approx0.9$ (p=0.286). When we allow for GxE effects ("strata = 1" gives the column in the data with information about strata) but not PoO effects, we get the following:

res3 <- haplinSlide(filename = "sim/sim1.dat", markers = 1,
 winlength = 1, reference = 1, n.vars = 1,
 allele.sep = " ", strata = 1)

##
## Running Haplin on Window '1' (1/1)...: done
##
## --- haplinSlide has completed ---

cbind(stratum=res3$`1`[3:6, "stratum"],
 signif(res3$`1`[3:6, c("RR.est.", "RR.p.value", "gxe_child_pval")], 3))

## stratum RR.est. RR.p.value gxe_child_pval
## 3 1 1.00 NA 0.281
## 4 1 1.14 0.295 0.281
## 5 2 1.00 NA 0.281
## 6 2 1.04 0.751 0.281

Now Haplin calculates the RR in each stratum (stratum 1: RR=1.14, p=0.295; stratum 2: RR=1.04, p=0.751), and gives a p-value for an overall GxE effect (p=0.281). Finally, we run the full PoOxE analysis.

res4 <- haplinSlide(filename = "sim/sim1.dat", markers = 1,
 winlength = 1, reference = 1, n.vars = 1,
 allele.sep = " ", poo = T, strata = 1)

##
## Running Haplin on Window '1' (1/1)...: done
##
## --- haplinSlide has completed ---

cbind(stratum=res4$`1`[3:6, 1],
 signif(res4$`1`[3:6, c("RRcm.est.", "RRcm.p.value", "RRcf.est.",
 "RRcf.p.value", "RRcm_RRcf.est.",
 "RRcm_RRcf.p.value", "gxe_poo_pval")], 3))

## stratum RRcm.est. RRcm.p.value RRcf.est. RRcf.p.value RRcm_RRcf.est.
## 3 1 1.000 NA 1.000 NA 1.000
## 4 1 0.397 2.38e-06 1.870 9.57e-07 0.212
## 5 2 1.000 NA 1.000 NA 1.000
## 6 2 1.650 1.36e-04 0.437 9.05e-06 3.770
## RRcm_RRcf.p.value gxe_poo_pval
## 3 NA 1.06e-29
## 4 0.00e+00 1.06e-29
## 5 NA 1.06e-29
## 6 4.96e-14 1.06e-29

Now we get not only a highly significant overall effect (p $\approx{10}^{-29}$), but we also detect the PoO effects in each stratum. The column RRcm_RRcf.est. gives the RRR for each stratum (stratum 1: RRR=0.397/1.870=0.212; stratum 2: RRR=1.650/0.437=3.770). Note that with a so-called qualitative interaction ("opposite" effects across strata), the RRR is magnified. This becomes even more pronounced for the overall analysis, where RRR=3.770/0.212=17.78.
